# Evolution and networks in ancient and widespread symbioses between Mucoromycotina and liverworts

**DOI:** 10.1007/s00572-019-00918-x

**Published:** 2019-11-13

**Authors:** William R. Rimington, Silvia Pressel, Jeffrey G. Duckett, Katie J. Field, Martin I. Bidartondo

**Affiliations:** 1 Department of Life Sciences, Imperial College London, London, SW7 2AZ UK; 2 Department of Life Sciences, Algae, Fungi and Plants Division, Natural History Museum, London, London, SW7 5BD UK; 3 Comparative Plant and Fungal Biology, Royal Botanic Gardens, Kew, Richmond, TW9 3DS UK; 4 Centre for Plant Sciences, Faculty of Biological Sciences, University of Leeds, Leeds, LS2 9JT UK

**Keywords:** Fine root endophytes, Glomeromycotina, Liverworts, Mucoromycotina, Networks, Terrestrialization

## Abstract

Like the majority of land plants, liverworts regularly form intimate symbioses with arbuscular mycorrhizal fungi (Glomeromycotina). Recent phylogenetic and physiological studies report that they also form intimate symbioses with Mucoromycotina fungi and that some of these, like those involving Glomeromycotina, represent nutritional mutualisms. To compare these symbioses, we carried out a global analysis of Mucoromycotina fungi in liverworts and other plants using species delimitation, ancestral reconstruction, and network analyses. We found that Mucoromycotina are more common and diverse symbionts of liverworts than previously thought, globally distributed, ancestral, and often co-occur with Glomeromycotina within plants. However, our results also suggest that the associations formed by Mucoromycotina fungi are fundamentally different because, unlike Glomeromycotina, they may have evolved multiple times and their symbiotic networks are un-nested (i.e., not forming nested subsets of species). We infer that the global Mucoromycotina symbiosis is evolutionarily and ecologically distinctive.

**Electronic supplementary material:**

The online version of this article (10.1007/s00572-019-00918-x) contains supplementary material, which is available to authorized users.

## Introduction

To what extent did fungi influence the conquest of land and greening of the planet by plants some 500 million years ago? This remains one of the most critical questions in land plant evolution ever since the idea of fungal-assisted plant terrestrialization was first proposed over 40 years ago (Pirozynski and Malloch 1975). This hypothesis posits that Precambrian green algae, the ancestors of land plants, were able to colonize land successfully by entering into partnerships with fungi. The fungi provided early rootless plants with nutrients and water in exchange for photosynthesis-derived carbohydrates. Following terrestrialization, this relationship evolved into mycorrhizal symbioses, now present in more than 85% of plants (Brundrett and Tedersoo 2018). Of these, arbuscular mycorrhizas (AM) formed by Glomeromycotina fungi are by far the most widespread, occurring in at least 72% of vascular plants (Brundrett and Tedersoo 2018).

Liverworts are one of the three groups of bryophytes, or non-vascular plants, alongside mosses and hornworts. Though the order of divergence of these groups remains under active debate (Puttick et al. 2018; Rensing 2018; de Sousa et al. 2019), bryophytes are generally regarded as some of the earliest terrestrial plants (Renzaglia et al. 2018) and have provided invaluable insights into the origin and evolution of key land plant innovations including mycorrhizas (e.g., Wang et al. 2010; Field et al. 2015a, b, 2016, 2019). Indeed, Glomeromycotina colonization in liverworts (Ligrone et al. 2007), together with the dominance of AM in extant land plant lineages, and their putative occurrence in fossils (Taylor et al. 1995) have long supported the paradigm that Glomeromycotina formed the ancestral plant-fungus symbiosis (Smith and Read 2008). However, this consensus, dating over 20 years, was challenged in 2011 by a proposal, using liverworts as a proxy for the first land plants, that Glomeromycotina-plant mutualism was predated by symbioses involving Endogonales belonging to Mucoromycotina (Bidartondo et al. 2011). Supporting physiological and phylogenetic evidence has since accumulated rapidly (Field et al. 2015a; Feijen et al. 2018; Hoysted et al. 2018). Mucoromycotina fungi have been shown to colonize the earliest-diverging liverwort clade, the Haplomitriopsida (Bidartondo et al. 2011), Koch’s postulates have been fulfilled, and isotope tracer experiments have demonstrated bidirectional nutritional exchange between some Haplomitriopsida and Mucoromycotina (Field et al. 2015b). Mucoromycotina fungi also colonize hornworts (Desirò et al. 2013) and the earliest-diverging vascular plants, lycophytes, and ferns (Rimington et al. 2015), indicating that these fungi are widespread symbionts of ancient plant lineages. Hornworts, lycophytes, and some non-Haplomitriopsida liverworts (Marchantiopsida and Pelliidae) are simultaneously colonized by both Mucoromycotina and Glomeromycotina; in liverworts, these “dual” colonizations have been shown to be more nutritionally beneficial than those involving only one fungal lineage (Field et al. 2016, 2019).

While the Glomeromycotina have been studied intensively for decades, the Mucoromycotina remain relatively poorly understood. Mucoromycotina and Glomeromycotina have recently been placed in the phylum Mucoromycota (Spatafora et al. 2016, but see Tedersoo et al. 2018), having been previously either an unplaced subphylum (Mucoromycotina) or a monophyletic phylum (Glomeromycota) (Schüßler et al. 2001; Hibbett et al. 2007). Of the three Mucoromycotina orders (Endogonales, Mucorales, and Umbelopsidales), only members of Endogonales (Endogonaceae and Densosporaceae) are known to enter into symbioses with plants (Desirò et al. 2017), being common endosymbionts of early-diverging lineages (liverworts, hornworts, lycophytes, and ferns) (Bidartondo et al. 2011; Desirò et al. 2013; Rimington et al. 2015) with some members ectomycorrhizal with trees (Walker 1985; Desirò et al. 2017; Yamamoto et al. 2017). Recent reports indicate that fine root endophytes, arbuscule-forming fungi found throughout vascular plants (Orchard et al. 2017a), may also be members of the Mucoromycotina rather than Glomeromycotina (Orchard et al. 2017b; Walker et al. 2018). Therefore, our understanding of the host range of Mucoromycotina across land plants and appreciation of their potential significance in modern ecosystems is expanding rapidly.

Here, we present a worldwide analysis of symbiotic Mucoromycotina associating with liverworts—the Haplomitriopsida, Marchantiopsida (complex thalloid), and Pelliidae (simple thalloid). Through DNA sequencing of 18S rDNA, species delimitation, and ancestral reconstruction, we aimed to shed light on this symbiosis by revealing its diversity and global distribution and comparing it to the symbiosis formed by Glomeromycotina (Rimington et al. 2018). We also present a first network analysis of fungal interactions with non-vascular plants. Network analysis allows visualization and quantification of how members of a network interact and it has become popular across biotic interactions, including mycorrhizas, to show which plants interact with which fungi, and vice versa, and to infer symbiotic ecology and evolution (Southworth et al. 2005; Bascompte and Jordano 2014; van der Heijden et al. 2015). Plant and fungal taxa represent nodes, and patterns of fungal occurrences are links connecting the two sets (fungi vs. plants or vice versa) of nodes. Once nodes and links are established, mycorrhizal network architecture can be quantified and compared.

## Methods

### Plant material and symbiotic fungi

We focused on liverwort clades (Haplomitriopsida, Marchantiopsida (complex thalloids), and Pelliidae (simple thalloids, within Jungermanniopsida)) with Mucoromycotina associations. The largest group of liverworts, the leafy Jungermanniidae, forms associations only with ascomycetes or basidiomycetes or lack fungi (Pressel et al. 2010). Liverwort collection sites were in 24 countries and all continents except Antarctica (Table S1). In total, 674 mature liverwort gametophytes were newly collected from the classes Haplomitriopsida (72 samples), Marchantiopsida (411), and Pelliidae (191). Specimen vouchers have been deposited at the Natural History Museum, London. Using the latest nomenclatures, samples were assigned to 85 species (Soderstrom et al. 2016; Stotler and Crandall-Stotler 2017). Only genus-level identification was possible for 49 samples, so the total number of species is likely higher than 85. Within 3 days of collection liverwort samples were cleaned of soil and debris using water and forceps. Then, the thallus midrib was dissected as this is where fungal colonization is highest (Pressel et al. 2010). Dissection was performed by removing the wings and rhizoids and cutting thallus sections ca. 3 mm^2^ that were placed in CTAB buffer and stored at − 20 °C.

Liverwort sections were used for sequencing the 18S ribosomal RNA gene of Mucoromycotina fungi. This gene was selected as it is the norm for investigating Mucoromycotina in plants (Bidartondo et al. 2011; Desirò et al. 2013; Rimington et al. 2015) and allows comparison with previous studies. The advantages and disadvantages of molecular detection using the 18S for endomycorrhizas have been reviewed by Öpik et al. (2014). Genomic DNA extraction was performed using chloroform extraction (Gardes and Bruns 1993) and the GeneClean II kit (QBioGene). After extraction, fungal DNA was amplified using the universal fungal primers NS1 (White et al. 1990) and EF3 (Smit et al. 1999) and JumpStart (Sigma) using the following PCR settings: 94 °C for 2 min; 34 cycles of 94 °C for 30 s, 53 °C for 30 s, and 72 °C for 1 min 30 s; 72 °C for 7 min. Following PCR, products were cloned with the Invitrogen TOPO TA Cloning Kit. We re-amplified DNA from between four and eight *E. coli* colonies per sample using JumpStart and NS1/EF3 with the following PCR settings: 94 °C for 7 min; 25 cycles of 94 °C for 30 s, 53 °C for 30 s, and 72 °C for 1 min 30 s; 72 °C for 5 min. The products of this second PCR were prepared for sequencing using ExoSAP-IT (Affymetrix) and BigDye v. 3.1 (Applied Biosystems) with the NS1 primer. For sequencing, an ABI3730 genetic analyzer was used (Applied Biosystems). The NCBI BLAST was used to assign DNA sequences (ca. 600 bp) to subphyla, and those found to be Mucoromycotina were further sequenced using the primers NS3 and NS5 (White et al. 1990). Sequences (ca. 1600 bp) were edited and assembled into contigs using Geneious v. 7 (Kearse et al. 2012). In cases where more than one clone from a sample was identified as Mucoromycotina, the sequences were aligned, and if they shared a pairwise similarity of more than 98%, then only one was selected for full sequencing of the 18S gene. The MUSCLE alignment algorithm (Edgar 2004) was used in MEGA (Kumar et al. 2016). The programs UCHIME (Edgar et al. 2011) and UNOISE2 (Edgar 2016) were used to test for chimeras.

### Phylogenetic analysis and species delimitation

The sequences produced in this investigation were combined with Mucoromycotina sequences from previous investigations of liverworts (Bidartondo et al. 2011; Field et al. 2015b, 2016), hornworts (Desirò et al. 2013), lycophytes, and ferns (Rimington et al. 2015), as well as sequences from Endogonales fruitbodies. Throughout these analyses, after the sequences were aligned, MEGA was used to test evolutionary models and for producing maximum likelihood phylogenies. Bayesian inference was performed using MrBayes (Huelsenbeck and Ronquist 2001) and outputs visualized using FigTree v. 1.4 (Rambaut 2012).

Two species delimitation methods, Poisson Tree Processes (PTP) and Generalized Mixed Yule Coalescent (GMYC), were used to group Mucoromycotina sequences into taxa. These methods are both superior to sequence-similarity OTU-calling methods as they incorporate phylogenetic analyses. Prior to these analyses, ALTER was used to remove haplotype sequences (Glez-Pena et al. 2010); haplotypes create zero branch lengths which can negatively influence analyses (Fujisawa and Barraclough 2013). The PTP is the simpler of the two methods and uses a rooted phylogenetic tree to model speciation rate (Zhang et al. 2013). This model utilizes the branch lengths of the tree with the assumption that each substitution creates a probability of speciation, as such, as the number of substitutions increases so does the probability of speciation. The input trees for this analysis were created using RAxML (Stamatakis 2014) which was utilized using RAxML-HPC2 on XSEDE on the CIPRES scientific gateway with 1000 bootstrap iterations (Miller et al. 2010). The PTP was performed using an updated version of the original analysis called mptp (Kapli et al. 2017) which is able to use Markov Chain Monte Carlo (MCMC) sampling (in all cases 10,000,000 generations were run) to produce support values for the groupings produced during delimitation. The GMYC method is more complex than PTP; it requires an unrooted, ultrametric, time-calibrated phylogenetic tree as input. The model then uses branch lengths to differentiate between within-population coalescence and speciation events (Fujisawa and Barraclough 2013). Input trees were produced using BEAST2 (Bouckaert et al. 2014) run through CIPRES. Priors were added to the BEAST analysis using BEAUTi (Drummond and Rambaut 2007). In all analyses, the molecular clock was “Relaxed Clock Log Normal” and the population model was “Birth Death.” The bModelTest was utilized to select the evolutionary model (Bouckaert and Drummond 2017). The number of generations in the MCMC chain was influenced by the size of the alignment and the convergence success and ranged between 10,000,000 and 200,000,000. Tree sampling frequency was determinant upon MCMC chain length to ensure a total of 1000 trees were sampled during the analysis. Tracer v1.6 was used to visualize the BEAST outputs (Rambaut et al. 2014). Convergence was deemed to be successful if all effective sample size values were greater than 200. If any were below this value, the BEAST analysis was run again using an increased chain length. TreeAnnotator was run on CIPRES to convert the 1000 trees into one consensus tree using a 10% burn-in, common ancestor heights, and maximum clade credibility as the analysis settings (Drummond and Rambaut 2007). This consensus tree was imported into RStudio (RStudio Team 2015) using the “rncl” package, after which GMYC was run using the “splits” package. Like mptp, “splits” has the capability to calculate the confidence in the delimited groups produced. Singletons are known to influence these analyses so in cases where singletons were delimited by GMYC, the full analysis was run again with singleton sequences removed from the alignment. The results of these delimitation methods were used to assign sequences to “early-diverging plant-Mucoromycotina taxa” (epMT) (i.e., fungi associated with early-diverging plants). When mptp and GMYC did not agree on the groupings, the group with the highest confidence level was selected. Species accumulation curves were produced using the “vegan” package within RStudio. Two curves were produced: one containing only epMT and the other containing both epMT and singletons. Extrapolation of the number of taxa was performed using the bootstrap method.

The epMT were combined with the epGT (early-diverging plant Glomeromycotina taxa) previously detected in these plants (Rimington et al. 2018) to allow comparison of Mucoromycotina and Glomeromycotina. The method of producing epGT was the same as used when assigning epMT so these results are directly comparable. The epGT were used in network analysis and ancestral reconstruction of fungal symbioses in liverworts.

### Ancestral reconstruction

The ancestral state of fungal symbioses in liverworts was reconstructed using Mesquite v. 3.31 (Maddison and Maddison 2017) with a representative of each liverwort genus sampled. Prior to ancestral reconstruction, a liverwort phylogenetic tree was produced using 26S and trnK-psbA spacer sequences from GenBank (Fig. S3). Where possible, these sequences represented the most frequently analyzed species of the genus in this investigation. For some genera, only genes from species not included in this investigation were available; however, as the purpose of the tree was to represent the phylogenetic placement of genera relative to each other, this was not considered important. There are no published sequences of the Pelliidae liverwort *Sewardiella* so sequences from its closest related genus, *Petalophyllum*, were used in the analyses. As these two genera are the only two members of the family Petalophyllaceae, this should not influence the topology. The moss *Takakia ceratophylla* was included as an outgroup. Following alignment in MEGA, Bayesian inference was performed using MrBayes with the nst = 6 model, invgamma rates, and 10,000,000 MCMC generations. The phylogenetic tree was imported into Mesquite and the ancestral reconstruction was performed separately for Mucoromycotina and Glomeromycotina. The liverwort genera were scored for presence or absence of fungi based on the DNA sequencing results of this study (Mucoromycotina) and the results of Rimington et al. (2018) (Glomeromycotina). Ancestral reconstruction was performed using maximum likelihood and maximum parsimony. The Asymmetry Likelihood Ratio Test in Mesquite determined the best model to use was the Markov 1-parameter model. The results of the analyses for both Mucoromycotina and Glomeromycotina were viewed simultaneously using the mirror tree window in Mesquite. A second liverwort phylogeny was produced for these analyses based on phylogenies that used five (Forrest et al. 2006) and eleven genetic markers (Flores et al. 2017). This was done because the original tree produced using 26S and the trnK-psbA spacer contained small topological differences from trees that used a larger number of genes. Starting with the original, “uncorrected” phylogeny, Mesquite’s branch moving tool was used to reorder branches based on the larger phylogenies while maintaining tree branch lengths. Ancestral reconstruction analyses were run again on this new tree using the same settings as before.

### Network analysis

Network analysis was performed on liverwort samples collected from the South Island of New Zealand, the most sampled location and with the largest number of country-specific epMT (taxa only found in one country and none of the other countries sampled in this study). The presence of epMT endemic to islands means that network analysis on a larger scale would not be valid. Network analysis consisted of four parts: (1) visualization, (2) connectance, (3) nestedness, and (4) modularity. For each of these, three separate analyses were performed, a network consisting of liverworts and both fungal lineages (combined-network) and two networks consisting of liverworts and only one fungal lineage (Glomeromycotina-only and Mucoromycotina-only). (1) The network was visualized in RStudio using “igraph” and the Fruchterman-Reingold layout. The degree function was used to relate node size to connection number while the weight function was used to relate connector thickness to the number of times the association was observed. (2) Connectance is the proportion of observed interactions out of the number that are theoretically possible and was calculated in RStudio using the “bipartite” package. (3) Nestedness measures the extent to which specialists in a network interact with generalists. A number of methods have been proposed to measure this character including Matrix Temperature (T), Nestedness measure based on Overlap and Decreasing Fills (NODF), and the Brualdi and Sanderson metric (BR). The most widely used nestedness metric is T which measures the extent to which the matrix departs from perfect nestedness, a value of 0° denotes a perfectly nested network and 100° is perfectly un-nested (Atmar and Patterson 1993). The NODF method calculates values of nestedness for rows and columns in a matrix by producing an average nestedness value from all combinations of pairs of rows and columns. These values are then combined to provide a value for the whole matrix (Almeida-Neto et al. 2008). The BR metric is a count of the smallest number of absences or presences which must be removed to create a perfectly nested matrix (Brualdi and Sanderson 1999). The NODF and BR are considered to be superior to T (Strona et al. 2014). Greater details of these concepts are reviewed in Ulrich et al. (2009). The programs NeD (T, NODF, and BR) and ANIHADO (T and NODF) were used to calculate these nestedness values (Guimarães and Guimarães 2006; Strona et al. 2014). For these calculations presence/absence matrices were used that were first ordered based on the sums of marginal rows and columns with the most common in the top left of the matrix, as is necessary in nestedness calculations (Ulrich et al. 2009). To assess the significance of the nestedness scores, null model replicates were run to allow comparisons. The CE null model was used (null model 2) in both NeD and ANIHADO with 999 random network replicates. In this model, the probability of a matrix cell being occupied is proportional to the row and column total (Bascompte et al. 2003). (4) Modularity detects the presence of modules in a network and the extent to which the presence of modules is a character of the network. Modules are groups of nodes that are more linked to each other than they are to other nodes or modules. In modularity analysis, nodes are grouped to maximize the number of links within modules and minimize the number of links between modules. NetCarto was used to detect modules by simulated annealing to maximize modularity and assign roles to nodes (Guimera and Amaral 2005) with an initial temperature of 10, an iteration factor of 1.0, and a 0.999 cooling factor. Modularity significance was tested within NetCarto using 100 randomizations (Guimera et al. 2004). The average modularity value and standard deviation of these randomizations were used to produce a one-way *Z* value for significance of modularity. NetCarto can also assign roles to nodes within modules by producing a value for participation coefficient (PC) and within-module relative degree (RD) for each node. The assigned roles (ultra-peripheral node, peripheral node, non-hub connector, or connector hub) were labeled specialists if PC ≤ 2.5 and RD ≤ 0.62 (Olesen et al. 2007). If PC > 2.5 and/or RD > 0.62, then the node was labeled a generalist.

## Results

### Mucoromycotina colonization is widespread in liverworts

Mucoromycotina were detected in 24% of the 674 liverwort samples and were found in sixteen countries and all six continents investigated. At the level of class/subclass colonization rates were Haplomitriopsida 69%, Marchantiopsida 14%, and Pelliidae 31%. Both Haplomitriopsida genera, *Treubia* and *Haplomitrium* (six species), six Marchantiopsida genera (13 species), and eight Pelliidae genera (17 species) were colonized by Mucoromycotina. Full results including fungal taxa detected and collection details are in Table S1. Results and detection rates for each liverwort group summarized at the genus level are in Table 1. Comparing our results with those of a worldwide analysis of Glomeromycotina in these plants (Rimington et al. 2018) indicates that many liverwort species can be colonized by both fungal lineages (Table 1), often simultaneously within the same plant individual (co-colonized). In total, 42 liverwort samples (15 Marchantiopsida, 27 Pelliidae), representing 21 species from 12 genera (six Marchantiopsida and six Pelliidae), were co-colonized by both Mucoromycotina and Glomeromycotina. Every Marchantiopsida genus harboring Mucoromycotina symbionts can be co-colonized by both fungal lineages. The Haplomitriopsida are the only group to form associations exclusively with Mucoromycotina. The most frequently co-colonized genus was *Fossombronia*, 22 samples belonging to eight species. The Mucoromycotina and Glomeromycotina that co-colonize liverworts are diverse and co-colonization is not limited to specific fungal taxa.

**Table 1:** Mucoromycotina detection in liverworts

		Sample no.	No. colonized by Mucoromycotina	Species no.	Colonized by Glomeromycotina?
All samples		674	165 (24%)	≥ 85	
Haplomitriopsida		72	50 (69%)	7	
Treubiales	Treubiaceae				
	*Treubia*	56	42 (75%)	2	No
Calobryales	Haplomitriaceae				
	*Haplomitrium*	16	8 (50%)	5	No
Marchantiopsida		411	56 (14%)	46	
Neohodgsoniales	Neohodgsoniaceae				
	*Neohodgsonia*	8	3 (38%)	1	Yes*
Lunulariales	Lunulariaceae				
	*Lunularia*	36	9 (25%)	1	Yes*
Marchantiales	Marchantiaceae				
	*Marchantia*	63	0	≥ 10	Yes
	*Preissia*	9	0	1	Yes
	Aytoniaceae				
	*Asterella*	81	24 (30%)	≥ 12	Yes*
	*Cryptomitrium*	6	0	2	No
	*Mannia*	5	0	≥ 1	Yes
	*Plagiochasma*	48	6 (13%)	≥ 2	Yes*
	*Reboulia*	9	0	1	Yes
	Cleveaceae				
	*Athalamia*	1	0	1	Yes
	*Clevea*	2	0	1	Yes
	*Sauteria*	2	0	1	No
	Conocephalaceae				
	*Conocephalum*	30	0	3	Yes
	Cyathodiaceae				
	*Cyathodium*	7	0	≥ 3	No
	Corsiniaceae				
	*Corsinia*	4	0	1	No
	Oxymitraceae				
	*Oxymitra*	1	0	1	No
	Targioniaceae				
	*Targionia*	34	10 (29%)	1	Yes*
	Monocleaceae				
	*Monoclea*	34	4 (12%)	2	Yes*
	Dumortieraceae				
	*Dumortiera*	31	0	1	Yes
Pelliidae		191	59 (31%)	≥ 32	
Pelliales	Noterocladaceae				
	*Noteroclada*	4	0	1	Yes
	Pelliaceae				
	*Pellia*	16	1 (6%)	3	Yes
Fossombroniales	Calyculariaceae				
	*Calycularia*	10	3 (30%)	1	Yes*
	*Sewardiella*	3	2 (67%)	1	Yes*
	Allisoniaceae				
	*Allisonia*	7	1 (14%)	1	Yes*
	Fossombroniaceae				
	*Fossombronia*	116	49 (42%)	≥ 15	Yes*
Pallaviciniales	Phyllothalliaceae				
	*Phyllothallia*	3	0	1	No
	Moerckiaceae				
	*Moerckia*	1	1 (100%)	1	Yes*
	Hymenophytaceae				
	*Hymenophyton*	4	0	1	Yes
	Pallaviciniaceae				
	*Jensenia*	1	0	1	Yes
	*Pallavicinia*	5	1 (20%)	1	Yes*
	*Podomitrium*	2	0	1	Yes
	*Symphyogyna*	19	1 (5%)	4	Yes

The number in brackets is the Mucoromycotina detection rate for the group/genus. The greater than or equal to symbol indicates some samples could only be identified to genus level, so it is the minimum number of species. Glomeromycotina colonization is based on Rimington et al. (2018). An asterisk indicates that individuals within the genus were found to be co-colonized by both fungal lineages

### Diverse Mucoromycotina taxa colonize early-diverging land plants

**Fig. 1:**
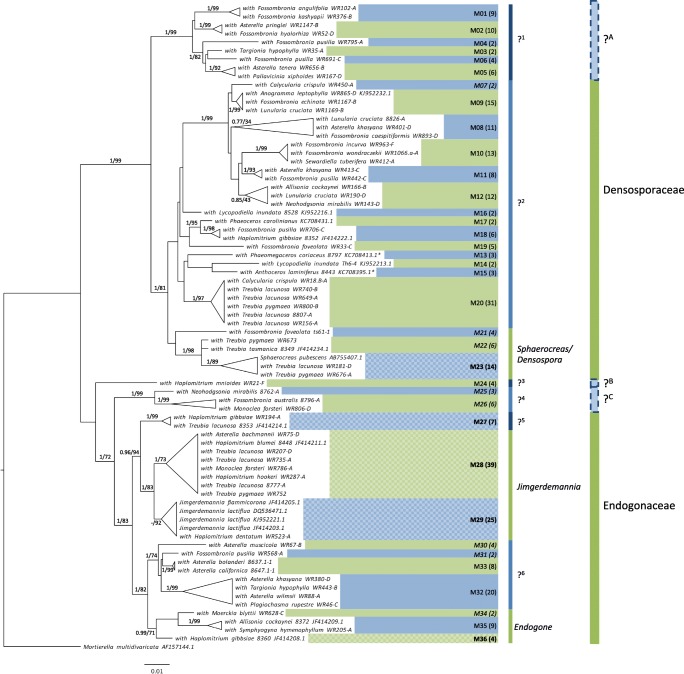
Diverse Mucoromycotina taxa colonize early-diverging plants. Maximum likelihood phylogeny of the Mucoromycotina that colonize liverworts and the results of species delimitation (epMT labels have been shortened to M) based on 18S DNA sequences. Support values are the result of both Bayesian inference and 1000 bootstrap replicates. Only support values for the main branches are provided—full support values and analysis settings are detailed in Fig. S1. A dash indicates Bayesian inference did not agree with maximum likelihood. Figures in brackets indicate the number of DNA sequences that belong to each epMT. The epMT in bold include sequences from Endogonales fruitbodies. Italicized epMT are specific to liverworts. Genus and family labels are based on Desirò et al. (2017). Question marks indicate putative new fungal genera (1–6) and families (A–C). Alternating blue and green are used to highlight different clades

Species delimitation using Generalized Mixed Yule Coalescent (GMYC) and multi-rate Poisson Tree Process (mPTP) placed the Mucoromycotina detected in liverworts, hornworts, lycophytes, and ferns into 36 taxa (epMT, early-diverging plant-Mucoromycotina taxa), summarized in Fig. 1 (see Fig. S1 for support values for this tree). Species accumulation curves indicate that 83–88% of the endosymbiotic Mucoromycotina that colonize the liverworts analyzed have been detected (Fig. S2). Eight of the 36 epMT are exclusive to liverworts, one to lycophytes, one to hornworts, and three to lycophytes and hornworts. Liverworts were colonized by more epMT (31 epMT) than hornworts (24 epMT), lycophytes (5 epMT), and ferns (2 epMT). Fifteen sequences were delimited as singletons; ten from liverworts, four from hornworts, and one from a lycophyte.

The majority of epMT have been assigned to the two Endogonales families (Endogonaceae and Densosporaceae). We detected at least six potentially new Endogonales genera, i.e., strongly supported clades in phylogenetic trees that do not contain representatives of any described Endogonales. There is little overlap between sequences of fruiting bodies and plant-colonizing Mucoromycotina. Only five of the epMT contained sequences from Mucoromycotina fruitbodies (epMT23, 27, 28, 29, and 36); the endosymbiotic Mucoromycotina that are members of these five epMT predominately originated from Haplomitriopsida liverworts, in particular *Haplomitrium*, or hornworts*.*

### Mucoromycotina formed the ancestral liverwort-fungal symbiosis

**Fig. 2:**
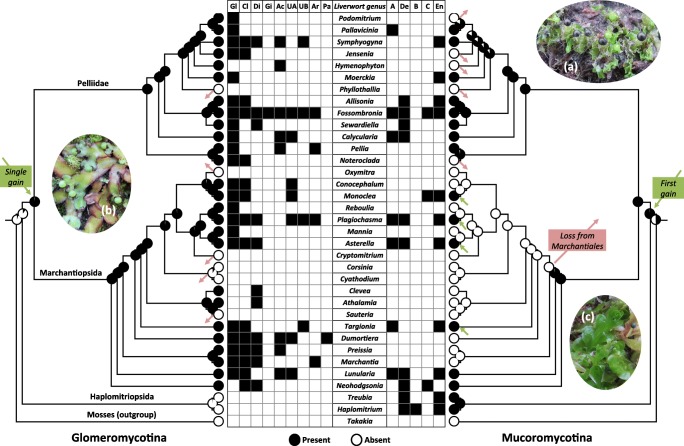
Ancestral reconstruction of fungal symbiosis in liverworts. The trees are mirror images of a Bayesian inference phylogeny produced using 26S, the trnK-psbA spacer, and the phylogenies of Forrest et al. (2006) and Flores et al. (2017). Ancestral reconstruction was performed using the Markov 1-parameter model. The grid indicates the presence of different Glomeromycotina and Mucoromycotina families. Family initials represent Gl, Glomeraceae; Cl, Claroideoglomeraceae; Di, Diversisporaceae; Gi, Gigasporaceae; Ac, Acaulosporaceae; UA, Undescribed Archaeosporales A; UB, Undescribed Archaeosporales B; Ar, Archaeosporaceae; Pa, Paraglomeraceae; A, Putative new Endogonales family A (Fig. 1); De, Densosporaceae; B, Putative new Endogonales family B; C, Putative new Endogonales family C; En, Endogonaceae. The gain and loss events are highlighted by green (gain) and pink (loss) arrows. See also Fig. S3. Examples of the different liverwort groups; (a) Pelliidae - *Fossombronia foveolata*, (b) Marchantiopsida - *Asterella australis*, (c) Haplomitriopsida - *Treubia pygmaea*. Note that the liverwort genus *Preissia* is now subsumed into *Marchantia* (Long et al. 2016)

Ancestral reconstruction using maximum likelihood analysis supports Mucoromycotina symbiosis as an ancestral state for all liverworts and that both Mucoromycotina and Glomeromycotina are ancestral symbionts for the non-Haplomitriopsida liverworts (Fig. 2). Maximum parsimony analysis also supports these ancestral states. There has been only one gain of Glomeromycotina during the evolution of liverworts, which occurred in the ancestor of all the non-Haplomitriopsida liverworts. This gain has been followed by five loss events, impacting six of the genera analyzed (*Phyllothallia*, *Oxymitra*, *Cryptomitrum*, *Corsinia*, *Cyathodium*, and *Sauteria*), but there have been no additional gains. On the other hand, Mucoromycotina symbiosis in liverworts has likely undergone a number of losses and reacquisitions. As well as the well-documented loss of fungal symbioses in Blasiales and Sphaerocarpales (Pressel et al. 2010), there appears to have been a major loss from the Marchantiales with a subsequent regain in four of the genera analyzed (*Targionia*, *Asterella*, *Plagiochasma*, and *Monoclea*). Mucoromycotina symbiosis appears to be the ancestral state for Pelliidae with losses in *Noteroclada*, *Phyllothalia*, *Hymenophyton*, *Jensenia*, and *Podomitrium*. Analysis of the “uncorrected” liverwort tree (Fig. S3), despite its slightly altered topology, supports identical ancestral states and losses and gains of both Glomeromycotina and Mucoromycotina. Presence/absence of fungal families in liverwort genera (Fig. 2) reveals that *Fossombronia* engages in the most diverse interactions with both fungal lineages. *Treubia* was only found to associate with two Mucoromycotina families, despite having the highest Mucoromycotina detection rate.

### Contrasting Mucoromycotina and Glomeromycotina networks

**Fig. 3:**
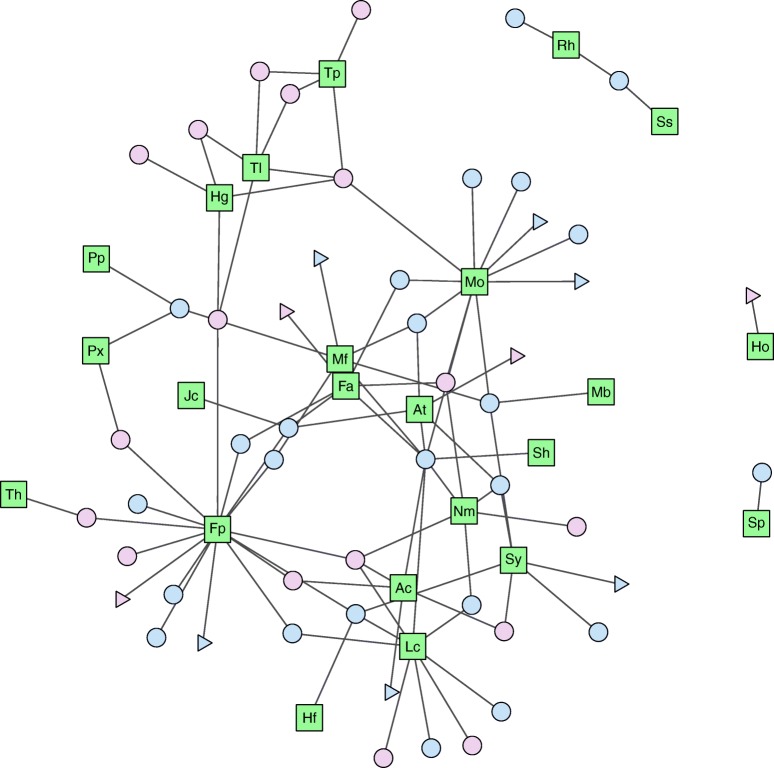
Network shared between liverworts of the South Island of New Zealand and Mucoromycotina and Glomeromycotina fungi. Green square nodes denote liverworts; blue and pink circles are Glomeromycotina and Mucoromycotina, respectively. Singletons are presented as triangles. Initials represent Ac, *Allisonia cockaynei*; At, *Asterella tenera*; Fa, *Fossombronia australis*; Fp, *Fossombronia pusilla*; Hg, *Haplomitrium gibbsiae*; Ho, *Haplomitrium ovalifolium*; Hf, *Hymenophyton flabellatum*; Jc, *Jensenia connivens*; Lc, *Lunularia cruciata*; Mb, *Marchantia berteroana*; Mf, *Marchantia foliacea*; Mo, *Monoclea forsteri*; Nm, *Neohodgsonia mirabilis*; Px, *Pallavicinia xiphoides*; Pp, *Podomitrium phyllanthus*; Rh, *Reboulia hemisphaerica*; Sh, *Symphyogyna hochstetteri*; Sy, *Symphyogyna hymenophyllum*; Sp, *Symphyogyna prolifera*; Ss, *Symphyogyna subsimplex*; Th, *Targionia hypophylla*; Tl, *Treubia lacunosa*; Tp, *Treubia pygmaea*. Labels for epMT and epGT are not included but can be seen in Fig. S4

The network shared between New Zealand (South Island) liverworts and Glomeromycotina and Mucoromycotina (combined-network) is shown in Fig. 3. Alternative visualizations, including Glomeromycotina- and Mucoromycotina-only networks, are shown in Fig. S4. The combined-network had a connectance of 8% while the Glomeromycotina- and Mucoromycotina-only networks each had a connectance of 11% and 13%, respectively. The different nestedness calculation methods did not always agree on whether the networks were nested (Table 2). The only consensus from all three methods was that the Mucoromycotina-only network is not nested. For the combined-network, NODF and T support the network is significantly nested. The Glomeromycotina-only network was supported as significantly nested by T and BR. Furthermore, when looking at the plants and fungi individually, the NODF method significantly supports that both the host and symbiont components of the network are nested in the combined- and Glomeromycotina-only networks. These results suggest that the combined- and Glomeromycotina-only networks are nested but the Mucoromycotina-only network is not. None of the three networks were found to be significantly modular (Table 2). The modularity analysis determined that the networks are dominated by specialists and not generalists, with specialists representing 86–94% of the nodes in the three networks (Table S2a). *Fossombronia pusilla* was the only connector hub in all three networks. *Monoclea forsteri* and *Lunularia cruciata* were connector hubs in the combined-network, likely due to their dual colonizations. The other generalist nodes identified (non-hub connectors) were both plants and fungi and depended upon the network in question. Detailed network analysis values are in Table S2a.

**Table 2:** Network analysis results

	Combined-network	Glomeromycotina-only	Mucoromycotina-only
No. liverwort species	23	18	14
No. fungal taxa (incl. singletons)	51	30	21
Total no. associations	148	69	79
Connectance	8%	11%	13%
Nestedness			
NODF_full_	14.68	18.36	13.44
Nested?	Yes (*p* = 0.018)	No (*p* = 0.087)	No (*p* = 0.23)
NODF_plants_	12.50	19.53	9.65
Nested?	Yes (*p* = 0.0065)	Yes (*p* = 0.0005)	No (*p* = 0.11)
NODF_fungi_	15.12	17.95	15.08
Nested?	Yes (*p* < 0.00001)	Yes (*p* = 0.0012)	No (*p* = 0.27)
T	17.8°	18.09°	31.45°
Nested?	Yes (*p* = 0.0069)	Yes (*p* = 0.0010)	No (*p* = 0.30)
BR	69	37	24
Nested?	No (*p* = 0.068)	Yes (*p* = 0.040)	No (*p* = 0.16)
Modularity			
No. modules	10	8	6
Modularity score	0.61	0.57	0.62
Significant?	No (*p* = 0.06)	No (*p* = 0.42)	No (*p* = 0.29)

The programs ANINHADO and NeD produced the same results for nestedness and significance so only the results of NeD are presented with the exception of NODF_full_, where the *p* value from ANINHADO is included, as this cannot be calculated by NeD

## Discussion

Mucoromycotina symbionts have been found throughout the early-diverging liverworts. Analysis of the networks shared between symbiotic fungi and their liverwort hosts suggests that Mucoromycotina symbiosis shares more similarities with ectomycorrhizas (ECM) than arbuscular mycorrhizas (AM).

## Diverse Mucoromycotina fungi colonize early-diverging land plants

Prior to this study nine liverwort species had been confirmed molecularly to host Mucoromycotina (Bidartondo et al. 2011). Our study has increased this number to 39. Furthermore, we have more than doubled the total number of plant species confirmed to enter into endosymbioses with Mucoromycotina: 39 liverworts, 15 hornworts (Desirò et al. 2013), four lycophytes, one fern (Rimington et al. 2015), and one angiosperm (Orchard et al. 2017b) though diverse vascular plants have been reported to harbor fine root endophytes (Orchard et al. 2017a). The Haplomitriopsida were the only plants exclusively colonized by Mucoromycotina, in line with previous reports (Bidartondo et al. 2011; Field et al. 2015b). All other liverwort genera found to associate with Mucoromycotina also enter into symbiosis with Glomeromycotina. The Mucoromycotina detected in Haplomitriopsida are generalists and of the nine epMT found in these liverworts, only one was Haplomitriopsida-specific. However, the Haplomitriopsida harbor a relatively low diversity of Mucoromycotina compared with the other liverwort groups—nine epMT vs. 15 in Marchantiopsida vs. 22 in Pelliidae. This pattern is maintained when sampling effort is taken into account; the number of epMT detected per colonized sample was 0.2 for Haplomitriopsida, 0.3 for Marchantiopsida, and 0.4 for Pelliidae. Despite also being colonized by Glomeromycotina, *Fossombronia* (Pelliidae) and *Asterella* (Marchantiopsida) were colonized by a larger number of epMT than *Treubia* and *Haplomitrium* combined. Thus, Haplomitriopsida exhibit specificity to a limited number of Mucoromycotina. It should be noted that in addition to Endogonaceae and Densosporaceae, the phylogenetic trees produced in this study, and by Desirò et al. (2017) when reclassifying the Endogonales, suggest there may be up to three additional families (Fig. 1).

Unlike Haplomitriopsida, Marchantiopsida and Pelliidae liverworts were regularly colonized by both Mucoromycotina and Glomeromycotina and we have increased the number of species confirmed to be colonized by both lineages from two (Field et al. 2016) to 28. This may provide a counterexample to a recent report that dual colonizations by different fungal lineages are rare and unstable (Werner et al. 2018); not only was dual colonization common, but also the ancestral positions of these symbionts (Fig. 2) indicate that these symbioses can be considered evolutionarily stable. In liverworts, co-colonization has been shown to be nutritionally more beneficial than colonization by only one lineage, which may explain why Mucoromycotina symbiosis has been maintained in these plants despite the global dominance of Glomeromycotina (Field et al. 2016, 2019). The Glomeromycotina found in co-colonized individuals were disproportionately members of the “older,” non-Glomeraceae families. We have previously shown that 36% of Glomeromycotina colonized liverworts harbor exclusively non-Glomeraceae (Rimington et al. 2018). However, this value increases to 54% when considering only co-colonized samples. This may hint towards co-colonization being an ancient phenomenon thus supporting the hypothesis that dual symbioses were utilized by early land plants (Field et al. 2015a).

## The origins of fungal symbiosis in liverworts

Ancestral reconstruction supports Mucoromycotina symbiosis evolving before Glomeromycotina symbiosis in liverworts. Given current uncertainties surrounding the order of divergence of the three bryophyte lineages (liverworts, hornworts, and mosses) and the monophyly of bryophytes (Puttick et al. 2018; de Sousa et al. 2019), it is not yet possible to confirm or otherwise refute the recent, novel hypothesis, based on multidisciplinary evidence, of Mucoromycotina having formed the ancestral plant-fungus symbiosis (Bidartondo et al. 2011; Field et al. 2015a; Feijen et al. 2018).

The reconstruction has also suggested only one gain of Glomeromycotina in liverworts. This was followed by some losses of symbiosis but never reversion to Glomeromycotina. This pattern of single gain followed by loss without reversion is mirrored by arbuscular mycorrhizal seed plants (Maherali et al. 2016) likely resulting from loss of symbiosis genes (Delaux et al. 2014). In early-diverging liverworts, as in seed plants, there must be selection pressure to maintain Glomeromycotina symbiosis. This pressure is not the same for Mucoromycotina symbiosis, which was lost and then gained at least four times (Fig. 2). The Mucoromycotina taxa colonizing liverwort genera that reverted to Mucoromycotina symbiosis are the same as those in plants that have maintained the symbiosis. Thus, these appear to be true reversions and not novel forms of Mucoromycotina symbiosis. Loss and regain of Mucoromycotina endosymbiosis has also occurred in hornworts (Desirò et al. 2013), ferns, and probably lycophytes (Rimington et al. 2015). In contrast, the taxa that form ECM in angiosperms are distinct from those that form endosymbiosis and represent a new form of symbiosis (Fig. S5). In all instances, the liverwort genera that have re-established Mucoromycotina symbiosis have maintained Glomeromycotina symbiosis and there have been no cases of Mucoromycotina being re-established in a non-symbiotic genus. This could stem from these plants likely utilizing the same or similar gene pathways during the establishment of both Mucoromycotina and Glomeromycotina symbiosis. In fact, three symbiosis (*sym*) genes from Haplomitriopsida liverworts (which exclusively enter into Mucoromycotina symbioses) rescue Glomeromycotina symbiosis in a *Medicago truncatula* mutant lacking these genes (Wang et al. 2010). To be maintained, *sym* genes must be functional or they would degrade as in non-symbiotic mosses (Wang et al. 2010). It cannot be ruled out that the *sym* genes have other, non-symbiotic functions (Bonfante and Selosse 2010), but the work of Wang et al. (2010) strongly supports that at least some of the same genes are used by plants for both Mucoromycotina and Glomeromycotina symbioses. This would explain how Mucoromycotina symbiosis can be re-established and why, if both types of symbiosis are lost, then neither can be re-established.

The widespread occurrence of Mucoromycotina symbiosis in early-diverging liverworts and nutrient exchange studies (Field et al. 2015b, 2016, 2019) indicate that this relationship can be beneficial to liverworts. It is unknown why Mucoromycotina symbiosis has been lost from some liverworts that have maintained Glomeromycotina symbiosis (Fig. 2). A possible evolutionary scenario for this is related to the higher levels of dual colonization recorded in Pelliidae compared with Marchantiopsida and a single loss of Mucoromycotina symbiosis. Early during the evolution of the Marchantiopsida ca. 196 MYA (Villarreal et al. 2016), there was a complete loss of Mucoromycotina symbiosis (Fig. 2). This was the only major loss of either Mucoromycotina or Glomeromycotina detected (Fig. 2) and resulted in the common ancestor of the largest early-diverging liverwort order (Marchantiales) not entering into symbiosis with Mucoromycotina. As such, for members of this order to form Mucoromycotina symbioses, they first needed to evolve mechanisms to re-establish the relationship. Subsequently, unlike Glomeromycotina, Mucoromycotina is limited to only a few Marchantiales genera. The Pelliidae has experienced no major loss of Mucoromycotina during diversification; therefore, the symbiosis is more widespread throughout the group than Marchantiopsida and dual colonization is more common. Additionally, the Pelliidae genera for which Mucoromycotina was not detected were sampled a limited number of times and colonization of Pelliidae liverworts by Mucoromycotina may be as common as it is for Glomeromycotina (Table 1). Thus, the absence of Mucoromycotina symbiosis from genera that enter into Glomeromycotina symbiosis may be solely due to a single loss of Mucoromycotina from the common ancestor of Marchantiales liverworts. The reason for this loss is unknown.

## Distinctive symbiotic networks

To our knowledge, this is the first time network analysis has been performed on Mucoromycotina and is also the first investigation of the symbiotic fungal networks of non-seed plants. When the associations of Mucoromycotina and Glomeromycotina were analyzed together (combined-network), the network appeared to have low connectance and significant nestedness. However, when the fungi were analyzed separately, the network analysis results appeared different; significant nestedness was recorded for the Glomeromycotina-only network but no nestedness was found in the Mucoromycotina-only network (Table 2). Mutualistic networks, such as those between plants and their animal pollinators and seed dispersers, are commonly nested (Bascompte et al. 2003; Bascompte and Jordano 2007; Thébault and Fontaine 2010); however, recent attempts at analyzing plant-fungal mutualistic networks have revealed considerable variation in their structure, depending on mycorrhizal type (Roy-Bolduc et al. 2016). While analyses of plant-AM networks showed significant nestedness (Chagnon et al. 2012; Montesinos-Navarro et al. 2012; Chen et al. 2017), those of ectomycorrhizal networks revealed these to be un-nested (Bahram et al. 2014; Roy-Bolduc et al. 2016). Thus, the absence of nestedness in our Mucoromycotina-only network is interesting, as it suggests that the networks shared by Mucoromycotina and liverworts may be more like ectomycorrhizal networks than arbuscular mycorrhizal networks. This notion coincides with latest isotope tracer experiments showing that Mucoromycotina, unlike Glomeromycotina, are able to transfer significant amounts of organic nitrogen to liverwort hosts, on a par with ECM (Field et al. 2019). Furthermore, unlike Glomeromycotina, some Mucoromycotina fungi can form ECM (Walker 1985; Yamamoto et al. 2017). The absence of nestedness indicates that Mucoromycotina symbioses in liverworts have greater plasticity than Glomeromycotina symbioses likely resulting from the different lifestyles of the fungal symbionts; Glomeromycotina are limited to obligate endo-biotrophy while Mucoromycotina have options including endo-biotrophy, ecto-biotrophy, and saprotrophy. These Mucoromycotina nutritional options, combined with the variable symbiotic status of liverworts, perhaps show that both the symbiont and host may “choose” whether to engage in symbiosis depending upon their growth conditions. For example, unlike all Glomeromycotina, some Mucoromycotina species are not restricted to an obligate lifestyle, so they have a readily available carbon source in the soil and there may be no benefit from entering into symbiosis with a plant, and vice versa for plants with ready access to mineral nutrients (as in vascular plants that are facultatively mycorrhizal with Glomeromycotina). This flexibility is not only a character of these organisms today but was also likely important during plant terrestrialization (Field et al. 2015a). It is worth pointing out that initial ancestral reconstruction analyses—albeit severely constrained by the availability and quality of data—suggest that Mucoromycotina fungi, unlike Glomeromycotina, switched trophic lifestyles (see Supplementary material, Fig. S5).

It is unlikely that the Mucoromycotina-only network contains too few species to produce significant nestedness, but to increase the number of species, we ran analyses again including New Zealand South Island data on Mucoromycotina and Glomeromycotina in hornworts from Desirò et al. (2013) (Table S2b). Species delimitation methods showed the Mucoromycotina that colonize hornworts are the same as those that colonize liverworts, so it is appropriate to combine these data. This increased the number of plant and fungal species to 49, but the network remained un-nested (Table S2b). As this number of species is higher than the number that supports significant nestedness in the Glomeromycotina-only network, we deduce that the lack of significance recorded is not the result of lower species number. Incidentally, when these data from hornworts were included in the Glomeromycotina-only and combined-networks, the support for nestedness was either the same or increased, further validating the use of these additional data. The connectance observed in the Glomeromycotina-only network was the same as previously recorded for a network between AM and flowering plants, suggesting that the liverwort network is functioning in a similar manner (Montesinos-Navarro et al. 2012). Ultimately, our analyses suggest that Mucoromycotina symbiosis with bryophytes should not be viewed as a type of arbuscular mycorrhiza formed by a fungal lineage different from Glomeromycotina, but as a distinctive symbiosis.

*Fossombronia pusilla* appeared as the most important member of all three networks and was the only connector hub in all three. Without *F. pusilla*, the network structures would have been different. This important network position stems from the ability of *Fossombronia* species to enter into the most diverse interactions with both Glomeromycotina and Mucoromycotina (Figs. 1 and 2). Furthermore, they were the most frequently co-colonized plants throughout this study. These results suggest that *Fossombronia* may be pivotal for conserving both Mucoromycotina and Glomeromycotina diversity. A likely explanation may be that the genus contains species ranging from fugitive ephemerals to those forming perennial colonies (Blockeel et al. 2014).

This pioneering study of the Mucoromycotina symbiosis has numerous caveats that are likely to affect all analyses. Here, we only mention some of the main caveats to qualify interpretation of our inferences and to inform subsequent studies. With increased sample sizes, variation in intraspecific colonization needs investigation because it may reflect detection problems and/or biological characteristics. For instance, plant-Mucoromycotina interactions may be facultative, except in Haplomitriopsida where their pervasiveness indicates those may be obligate. Small sample sizes, sampling unevenness, and small network dimensions in particular may affect network analysis differently, e.g., regarding nestedness and connectance and these should all be addressed. There is a need for improved calibrations for species delineation analysis and intraspecific DNA sequence variation thresholds for fungi, and testing for the presence of cryptic plant species (e.g., for network hub species), as well as replicating network analysis outside New Zealand. The symbiotic status of all early nodes in plant evolution needs assessment to better infer the status of the most recent common ancestor of plants, and the phylogeny and trophic status of most Endogonales also merit analysis. More complex evolutionary models may potentially quantify variation in loss and gain rates across phylogeny and correlated evolution analysis may inform whether there has been continued co-occurrence of Endogonales and Glomeromycotina symbionts.

## Conclusions

We have shown that Mucoromycotina enter into evolutionarily and geographically widespread symbioses, like Glomeromycotina. However, two important differences emerged. Mucoromycotina symbioses are ancestral and may have evolved multiple times in liverworts, as in hornworts, unlike Glomeromycotina symbioses. Glomeromycotina networks are significantly nested, unlike Mucoromycotina ones. The genetic underpinnings and ecological implications of our findings now merit investigation.

## Electronic supplementary material

ESM 1 (DOCX 2369 kb)

ESM 2 (XLSX 66 kb)

ESM 3 (XLSX 57 kb)

## Data Availability

The DNA sequences produced have been uploaded to GenBank and have the following accession numbers: MH174461-MH174649.
