# Supplementary material for: Evolution and networks in ancient and widespread symbioses between Mucoromycotina and liverworts
Source: Mycorrhiza. 2019 Nov 13;29(6):551–65. doi: 10.1007/s00572-019-00918-x (PMC6890582; doi:10.1007/s00572-019-00918-x)
Supplement: Supplementary file 1 — (DOCX 2369 kb) [file 572_2019_918_MOESM1_ESM.docx]

**Supplementary material**

**Evolution and networks in ancient and widespread symbioses between Mucoromycotina and liverworts.** *Mycorrhiza*

William R. Rimington*^1,2,3^, Silvia Pressel^2^, Jeffrey G. Duckett^2^, Katie J. Field^4^, Martin I. Bidartondo*^1,3^

^1^Department of Life Sciences. Imperial College London. London, SW7 2AZ, UK.

^2^Department of Life Sciences, Algae, Fungi and Plants Division. Natural History Museum, London. London, SW7 5BD, UK.

^3^Comparative Plant and Fungal Biology, Royal Botanic Gardens, Kew. Richmond, TW9 3DS, UK.

^4^Centre for Plant Sciences, Faculty of Biological Sciences. University of Leeds. Leeds, LS2 9JT, UK.

*Corresponding authors: w.rimington@gmail.com, m.bidartondo@imperial.ac.uk

**Contents**

Figures S1-S5

Tables S1-S3

Discussion of initial ancestral Mucoromycotina lifestyle reconstructions

**Figures S1-S5**

**Fig. S1. Support values for phylogenetic tree in Fig. 1.** These phylogenetic trees include the same DNA sequences as those used to produce Fig. 1. (a) Bayesian inference phylogeny produced using MrBayes using the nst=2 model and invgamma rates run for 10,000,000 generations. (b) Maximum likelihood phylogeny produced using MEGA with the TN93 model, invariant gamma rates and 1,000 bootstrap replicates.

(a)

(b)

**Fig. S2. Species accumulation curves of Mucoromycotina that colonize early-diverging liverworts.** In red are the results of using epMT-only, in blue are the results of also including singletons in the analysis. Highlighted areas show the confidence intervals of 1,000 random permutations.

**Fig. S3. Ancestral reconstruction of fungal symbiosis in liverwort genera analyzed using ‘uncorrected’ phylogeny.** Both trees in this figure are identical and are the same as those in Fig. 2 except the topologies have not been edited to match the phylogenies of Forrest *et al.* (2006) and Flores *et al.* (2017). Ancestral reconstruction used the Markov 1-parameter model. The table provides the GenBank accession numbers of the sequences used to produce the phylogeny seen here and in Fig. 2.

**Fig. S4. Network shared between liverworts of the South Island of New Zealand, Mucoromycotina and Glomeromycotina fungi, related to Fig. 3.** Node size and connector thickness are both related to the number of connections on a log scale. Node size is related to the number of connectors, so the larger the node, the greater the importance to the network structure. Connector thickness is related to the number of those connections observed, so the thicker the line the more frequently it was detected. (a) Both fungal lineages, (b) Glomeromycotina-only, (c) Mucoromycotina-only. Green squares - liverworts, blue circles - Glomeromycotina, pink circles - Mucoromycotina. Initials represent: Ac - *Allisonia cockaynei*; At - *Asterella tenera*; Fa - *Fossombronia australis*; Fp - *Fossombronia pusilla*; Hg - *Haplomitrium gibbsiae*; Ho - *Haplomitrium ovalifolium*; Hf - *Hymenophyton flabellatum*; Jc - *Jensenia connivens*; Lc - *Lunularia cruciata*; Mb - *Marchantia berteroana*; Mf - *Marchantia foliacea*; Mo - *Monoclea forsteri*; Nm - *Neohodgsonia mirabilis*; Px - *Pallavicinia xiphoides*; Pp - *Podomitrium phyllanthus*; Rh - *Reboulia hemisphaerica*; Sh - *Symphyogyna hochstetteri*; Sy - *Symphyogyna hymenophyllum*; Sp - *Symphyogyna prolifera*; Ss - *Symphyogyna subsimplex*; Th - *Targionia hypophylla*; Tl - *Treubia lacunosa*; Tp - *Treubia pygmaea*. The epGT and epMT are represented by G and M, respectively. Singletons are represented by GS (Glomeromycotina) and MS (Mucoromycotina).

(a) Combined-network


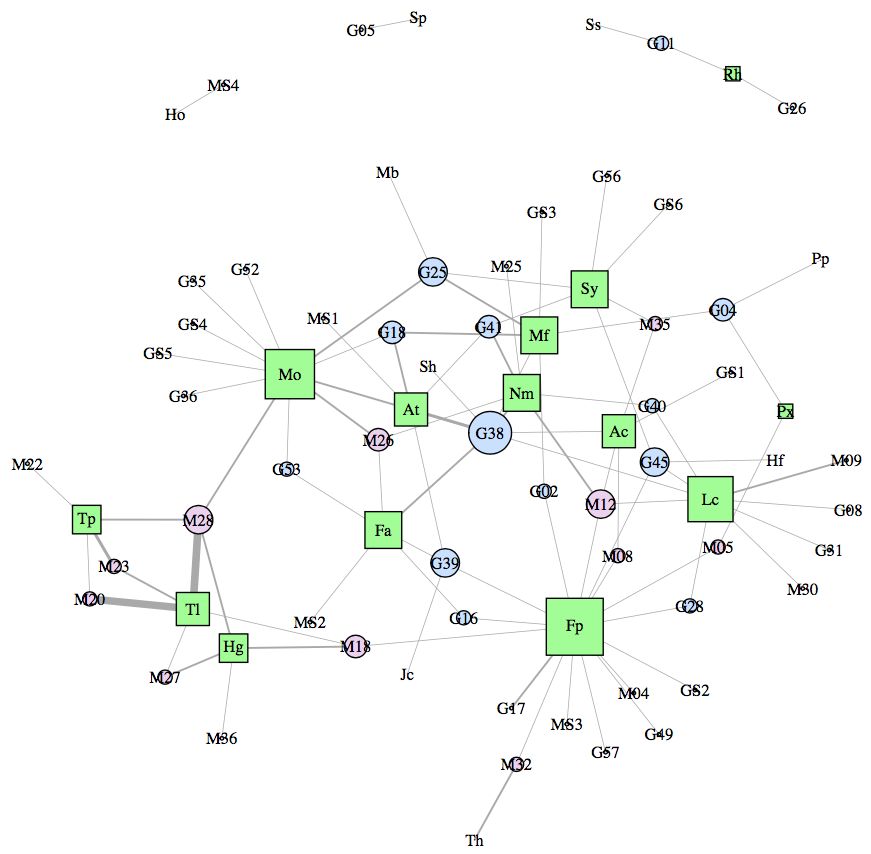


(b) Glomeromycotina- only


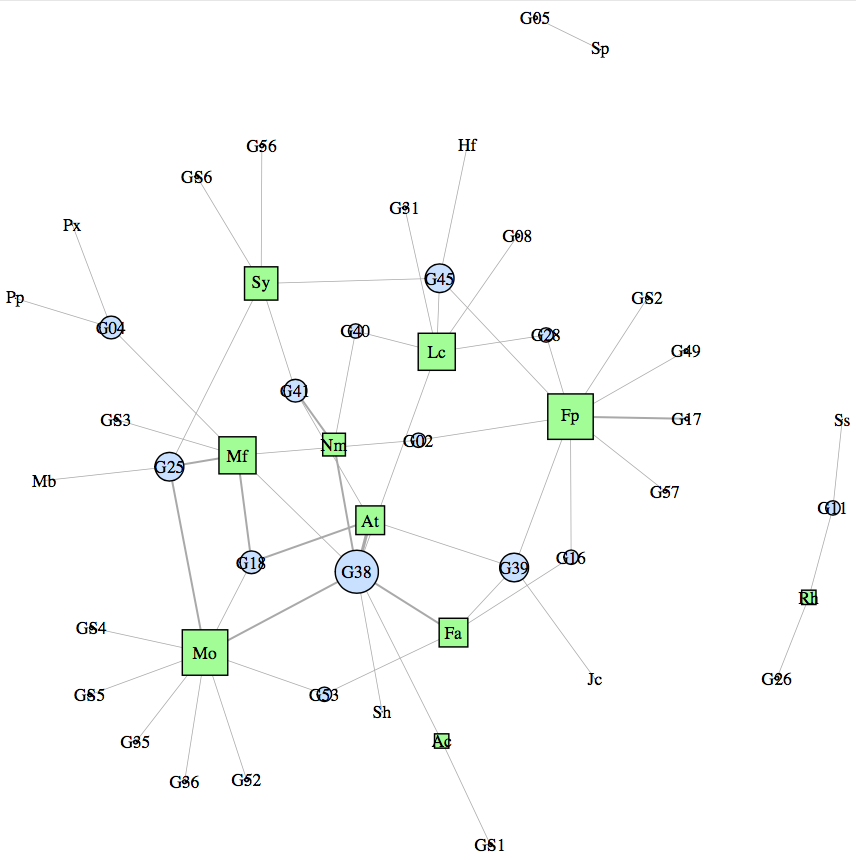


(c) Mucoromycotina- only


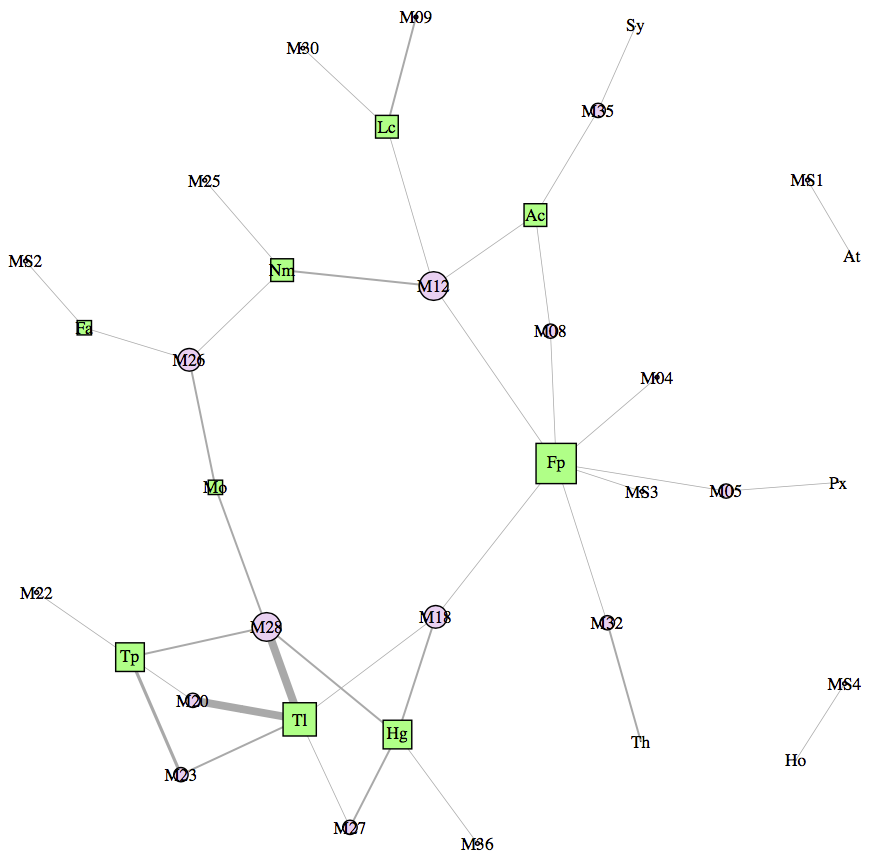


**Fig. S5. Ancestral reconstruction of Endogonales lifestyle.** Maximum likelihood phylogeny of Endogonales 18S sequences. Terminal branch color represents the assigned lifestyle (Table S3) while internal branch color and nodes represent the predicted ancestral lifestyles. Lifestyle abbreviations for sequences from plants: ECM - ectomycorrhizal, END - endosymbiotic. Abbreviations for fruitbody sequences: SAP - saprotrophic, SAP/ECM - saprotrophic or ectomycorrhizal, SAP/END - saprotrophic or endosymbiotic, UKN/END - unknown but cluster with endosymbiotic sequences. Tree rooted on saprotrophic Mortierellomycotina.

**Tables S1-S3**

**Table S1. Liverwort samples analyzed, collection details and Mucoromycotina epMT detected.**  Some samples could only be confidently identified to genus level so are labeled ‘sp.’. Samples in bold were simultaneously colonized by both Mucoromycotina and Glomeromycotina - the Glomeromycotina results are from Rimington *et al.* (2018). Collectors were as follows: DJR - David Read, JGD - Jeffrey G Duckett, KJF - Katie J Field, MIB - Martin I Bidartondo, SP - Silvia Pressel, WRR - William R Rimington.

Provided as a separate excel file.

**Table S2. a) Full results of network analysis.** For nestedness, only the results of NeD are presented, except NODF_full_, which is from ANINHADO. The percentage in brackets for role identify is the proportion of nodes in the network that were assigned that role; **b)** Network analysis results including New Zealand South Island data on Mucoromycotina and Glomeromycotina in hornworts from Desirò *et al.* (2013).

**a)**

|  | **Combined-network** | **Glomeromycotina-only** | **Mucoromycotina-only** |
| --- | --- | --- | --- |
| Number of liverwort species | 23 | 18 | 14 |
| Number of fungal taxa (including singletons) | 51 | 30 | 21 |
| Total number of associations | 148 | 69 | 79 |
| Number of associations in binary matrix | 96 | 58 | 38 |
|  |  |  |  |
| **Connectance** | 8% | 11% | 13% |
|  |  |  |  |
| **Nestedness** |  |  |  |
| NODF |  |  |  |
| NODF_full_ | 14.68 | 18.36 | 13.44 |
| Average nestedness of random networks | 11.21 | 14.64 | 16.09 |
| Std dev of nestedness of random networks | 1.66 | 2.73 | 3.61 |
| Z-score | 2.09 | 1.36 | 0.73 |
| p-value | 0.0184 | 0.0869 | 0.2318 |
| Significantly nested? | Yes (p<0.05) | No (p>0.05) | No (p>0.05) |
|  |  |  |  |
| NODF_plants_ | 12.50 | 19.53 | 9.65 |
| Average nestedness of random networks | 9.085 | 12.27 | 13.029 |
| Std dev of nestedness of random networks | 1.376 | 2.216 | 2.788 |
| Z-score | 2.482 | 3.277 | 1.211 |
| p-value | 0.0065 | 0.0005 | 0.1129 |
| Significantly nested? | Yes (p<0.01) | Yes (p<0.001) | No (p>0.05) |
|  |  |  |  |
| NODF_fungi_ | 15.12 | 17.95 | 15.08 |
| Average nestedness of random networks | 9.978 | 12.77 | 13.75 |
| Std dev of nestedness of random networks | 1.024 | 1.699 | 2.206 |
| Z-score | 5.02 | 3.048 | 0.602 |
| p-value | < 0.00001 | 0.0012 | 0.2736 |
| Significantly nested? | Yes (p<0.001) | Yes (p<0.01) | No (p>0.05) |
|  |  |  |  |
| T | 17.80 | 18.09 | 31.45 |
| Average nestedness of random networks | 23.002 | 27.538 | 33.662 |
| Std dev of nestedness of random networks | 2.109 | 3.069 | 4.194 |
| Z-score | 2.465 | 3.078 | 0.528 |
| p-value | 0.0069 | 0.0010 | 0.2988 |
| Significantly nested? | Yes (p<0.01) | Yes (p<0.01) | No (p>0.05) |
|  |  |  |  |
| BR | 69 | 37 | 24 |
| Average nestedness of random networks | 73.243 | 41.03 | 25.88 |
| Std dev of nestedness of random networks | 2.84 | 2.304 | 1.877 |
| Z-score | 1.494 | 1.749 | 1.002 |
| p-value | 0.0676 | 0.0401 | 0.1582 |
| Significantly nested? | No (p>0.05) | Yes (p<0.05) | No (p>0.05) |
|  |  |  |  |
| **Modularity** |  |  |  |
| Number of modules | 10 | 8 | 6 |
| Average number of nodes per module (and range) | 7.4 (2-13) | 6 (2-9) | 5.8 (2-10) |
| Network modularity score | 0.61 | 0.57 | 0.62 |
| Average modularity of random networks | 0.58 | 0.57 | 0.61 |
| Std dev of modularity of random networks | 0.01 | 0.02 | 0.02 |
| Z-score | 1.55 | 0.21 | 0.54 |
| p-value | 0.06 | 0.42 | 0.29 |
| Significantly modular? | No (p>0.05) | No (p>0.05) | No (p>0.05) |
|  |  |  |  |
| Roles |  |  |  |
| Specialists | 65 (88%) | 41 (85%) | 33 (94%) |
| Number of Ultra-peripheral nodes | 49 (66%) | 30 (63%) | 27 (77%) |
| Number of Peripheral nodes | 16 (22%) | 11 (23%) | 6 (17%) |
|  |  |  |  |
| Generalists | 9 (12%) | 7 (15%) | 2 (6%) |
| Number of Non-hub connectors | 6 (8%) | 6 (13%) | 1 (3%) |
| Number of Connector hubs | 3 (4%) | 1 (2%) | 1 (3%) |

**b)**

|  | **Combined-network** | **Glomeromycotina-only** | **Mucoromycotina-only** |
| --- | --- | --- | --- |
| No. liverwort species | 23 | 18 | 14 |
| No. hornwort species | 5 | 3 | 5 |
| No. fungal taxa (incl. singletons) | 60 | 29 | 31 |
| Total no. associations | 186 | 81 | 106 |
|  |  |  |  |
| **Connectance** | 8% | 10% | 10% |
|  |  |  |  |
| **Nestedness** |  |  |  |
| NODF_full_ | 13.83 | 17.78 | 14.45 |
| NODF_plants_ | 12.41 | 18.65 | 12.23 |
| Nested? | Yes (p<0.01) | Yes (p<0.001) | No (p>0.05) |
| NODF_fungi_ | 14.13 | 17.41 | 15.48 |
| Nested? | Yes (p<0.001) | Yes (p<0.01) | Yes (p<0.05) |
|  |  |  |  |
| T | 15.9° | 19.8° | 24° |
| Nested? | Yes (p<0.01) | Yes (p<0.05) | No (p>0.05) |
|  |  |  |  |
| BR | 92 | 44 | 41 |
| Nested? | Yes (p<0.05) | Yes (p<0.05) | No (p>0.05) |
|  |  |  |  |
| **Modularity** |  |  |  |
| No. modules | 9 | 8 | 9 |
| Modularity score | 0.57 | 0.56 | 0.56 |
| Significant? | No (p>0.05) | No (p>0.05) | No (p>0.05) |

**Table S3. Lifestyles assigned to Mucoromycotina sequences.** List of sequences used to produce Fig. S5 (including accession numbers where appropriate) and their lifestyles. Lifestyle initials are as follows: ECM - ectomycorrhizal (sequences reported from three roots and *Jimgerdemannia* fruitbodies (Desirò et al. 2017), END - endosymbiotic (all sequences from liverworts, hornworts, lycopods, ferns and fine root endophytes), SAP - saprotrophic (the majority of *Endogone* fruitbodies, Desirò et al. 2017), SAP/ECM - saprotrophic or ectomycorrhizal (probably saprotrophic but with one report of ectomycorrhizal status, Yamamoto et al. 2017), SAP/END - saprotrophic or endosymbiotic (fruitbodies for which lifestyle is yet to be confirmed and could be either saprotrophic or endosymbiotic, Hirose et al. 2014), UKN/END - unknown lifestyle but sequence clusters with endosymbiotic sequences (fruitbodies for which lifestyle has not been reported but cluster with endosymbiotic sequences). Those in bold originated from fruitbodies while those not in bold originated from sequencing of plant material.

Provided as a separate Excel file.

**Ancestral reconstructions of Mucoromycotina lifestyles**

Here we detail our initial ancestral reconstruction analyses that investigated the trophic lifestyles of Mucoromycotina (Endogonales) fungi.

**Methods**

The lifestyles of Mucoromycotina in the order Endogonales were mapped onto a phylogenetic tree to allow ancestral reconstruction. The phylogenetic tree was produced using the 400 available 18S sequences for Endogonales, including the liverwort sequences produced during this study as well as those previously published from liverworts (Bidartondo et al. 2011; Field et al. 2015, 2016), hornworts (Bidartondo et al. 2011; Desirò et al. 2013), lycopods (Rimington et al. 2015) and ferns (Bidartondo et al. 2011; Rimington et al. 2015). The tree also included Mucoromycotina sequences reported as being fine root endophytes (Orchard et al. 2017) and ectomycorrhizal (Tedersoo et al. 2008; Yamamoto et al. 2017). Finally, all the available Endogonales fruitbody sequences were included (Desirò et al. 2017), with *Mortierella multidivaricata* as an outgroup. Sequences were acquired from GenBank and UNITE (Kõljalg et al. 2013), aligned in MEGA using MUSCLE, and a maximum likelihood phylogeny was produced with RAxML, using the same settings as when producing trees for PTP species delimitation (see methods of main article).

The following three lifestyles were confidently assigned to the majority of sequences: endosymbiotic (all those from liverworts, hornworts, lycopods, ferns and fine root endophytes), ectomycorrhizal (ECM sequences and fruitbodies of the genus *Jimgerdemannia* (Desirò et al. 2017) and saprotrophic (the majority of *Endogone* fruitbodies). Three additional states were used for some fruitbody sequences as the lifestyle is not confirmed (Desirò et al. 2017): saprotrophic or ectomycorrhizal, saprotrophic or endosymbiotic, and unknown but potentially endosymbiotic. The assigned lifestyles for each sequence are in Table S3. Lifestyles were mapped onto the phylogeny using ‘phytools’ within RStudio (Revell 2011). Stochastic character mapping ancestral reconstruction was performed using SIMMAP (Bollback 2006) run within phytools and reconstruction was replicated 100 times using the ER model.

**Results**

The common ancestor of Endogonales was endosymbiotic (Fig. S5). The number of endosymbiosis losses and gains of an ectomycorrhizal or saprotrophic lifestyle is unclear due to the difficultly in assigning lifestyle status to some fruitbodies (Table S3). There has been only one clear loss of symbiosis and conversion to saprotrophy, within *Endogone*. There appear to have been at least two or three separate evolutions of an ectomycorrhizal lifestyle - in line with a previous prediction (Tedersoo and Smith 2013) - two originating from endosymbiosis and one potentially from saprotrophy.

**Discussion**

The different lifestyles of Mucoromycotina are distinct (Fig. S5), with effectively no crossover between endosymbiotic and non-endosymbiotic lineages. Only five epMT (epMT23, 27, 28, 29 and 36) contained fruitbody sequences and the remaining 31 taxa exclusively contained fungal DNA sequences from plants. These five taxa can be categorized as follows:

1) epMT27, 28 and 29 - These three taxa contained both endosymbiotic sequences (predominately from Haplomitriopsida liverworts) and fruitbodies sequences of supposedly ectomycorrhizal *Jimgerdemannia* fungi (Desirò et al. 2017);

2) epMT36 - This taxon primarily consists of sequences from saprotrophic *Endogone* but also contains a sequence originating from *Haplomitrium gibbsiae*;

3) epMT23 - This taxon is dominated by Haplomitriopsida sequences from endosymbionts but also contains sequences from *Sphaerocreas pubescens* (Hirose et al. 2014). It is unknown whether these fruitbodies are sexual endosymbionts or saproptrophs (Desirò et al. 2017).

The five fruitbody-containing epMT indicate that the endosymbiotic/non-endosymbiotic lifestyles are not mutually exclusive but the rarity of these crossovers suggests that the three major lifestyles of these fungi are not available to every Endogonales taxon. The axenic culturability of endosymbiotic Mucoromycotina shows that at least some taxa are not obligate biotrophs that are dependent upon their hosts and they can be ‘free-living’ facultative symbionts (Field et al. 2015; Pressel unpublished data). The absence of sequences from ‘free-living’, potentially asexual (therefore fruitbodies are not produced), Mucoromycotina that can form endosymbioses results from the identification and sequencing methods used, which have only utilized either plant material or fruitbodies. An alternative scenario is that the endosymbiotic Mucoromycotina that have been axenically cultured (Field et al. 2015; Pressel unpublished data) may be an exception and the majority of endosymbiotic Mucoromycotina may be asexual obligate biotrophs. The extent to which the different Endogonales taxa are fixed in their lifestyles and whether all endosymbiotic Mucoromycotina are facultative symbionts merit further investigation.

The ancestor of Endogonales most likely entered into endosymbioses with plants (Fig. S5). It appears that endosymbiosis has only been lost (and replaced by ECM or saprotrophy) and not been regained. A gain of saprotrophy at the expense of endosymbiosis is likely to have only occurred once. Within this lineage, ECM may have evolved once. It remains to be seen whether *Endogone pisiformis*/*aggregata* is a saprotrophic or ectomycorrhizal lineage. Looking at the lifestyles of these fungi (Fig. S5) it may be a report of *E. pisiformis* forming ECM (Yamamoto et al. 2017) is incorrect. If this were the case the entire *Endogone* clade would be saprotrophic and ECM only evolved on two occasions, from endosymbiosis. The changes in lifestyle from endosymbiosis to ECM or saprotrophy are limited to only three lineages. This further supports that endosymbiosis is ancestral. The variety of different lifestyle changes and the number of species that have changed lifestyle in Mucoromycotina are in contrast to Glomeromycotina - there is only one known species that has switched from AM (*Geosiphon pyriformis*) (Schüßler 2012).

**References**

Bidartondo MI, Read DJ, Trappe JM, Merckx V, Ligrone R, Duckett JG (2011) The dawn of symbiosis between plants and fungi. Biol Lett 7:574-577

Bollback JP (2006) SIMMAP: stochastic character mapping of discrete traits on phylogenies. BMC Bioinformatics 7:88

Desirò A, Duckett JG, Pressel S, Villarreal JC, Bidartondo MI (2013) Fungal symbioses in hornworts: a chequered history. Proc R Soc Lond B Biol Sci 280:20130207

Desirò A, Rimington WR, Jacob A, Pol NV, Smith ME, Trappe JM, Bidartondo MI, Bonito G (2017) Multigene phylogeny of Endogonales, an early diverging lineage of fungi associated with plants. IMA Fungus 8:245-257

Field KJ, Rimington WR, Bidartondo MI, Allinson KE, Beerling DJ, Cameron DD, Duckett JG, Leake JR, Pressel S (2015) First evidence of mutualism between ancient plant lineages (Haplomitriopsida liverworts) and Mucoromycotina fungi and its response to simulated Palaeozoic changes in atmospheric CO_2_. New Phytol 205:743-756

Field KJ, Rimington WR, Bidartondo MI, Allinson KE, Beerling DJ, Cameron DD, Duckett JG, Leake JR, Pressel S (2016) Functional analysis of liverworts in dual symbiosis with Glomeromycota and Mucoromycotina fungi under a simulated Palaeozoic CO_2_ decline. ISME J 10:1514-1526

Flores JR, Catalano SA, Muñoz J, Suárez GM (2017) Combined phylogenetic analysis of the subclass Marchantiidae (Marchantiophyta): towards a robustly diagnosed classification. Cladistics 34:517-541

Forrest LL, Davis EC, Long DG, Crandall-Stotler BJ, Clark A, Hollingsworth ML (2006) Unraveling the evolutionary history of the liverworts (Marchantiophyta): multiple taxa, genomes and analyses. Bryologist 109:303-334

Hirose D, Degawa Y, Yamamoto K, Yamada A (2014) *Sphaerocreas pubescens* is a member of the Mucoromycotina closely related to fungi associated with liverworts and hornworts. Mycoscience 55:221-226

Kõljalg U, Nilsson RH, Abarenkov K, Tedersoo L, Taylor AFS, Bahram M, Bates ST, Bruns TD, Bengtsson-Palme J, Callaghan TM, et al. (2013) Towards a unified paradigm for sequence-based identification of fungi. Mol Ecol 22:5271-5277

Orchard S, Hilton S, Bending GD, Dickie IA, Standish RJ, Gleeson DB, Jeffery RP, Powell JR, Walker C, Bass D, et al. (2017) Fine endophytes (*Glomus tenue*) are related to Mucoromycotina, not Glomeromycota. New Phytol 213:481-486

Revell LJ (2011) Phytools: an R package for phylogenetic comparative biology (and other things). Methods Ecol Evol 3:217-223

Rimington WR, Pressel S, Duckett JG, Bidartondo MI (2015) Fungal associations of basal vascular plants: reopening a closed book? New Phytol 205:1394-1398

Rimington WR, Pressel S, Duckett JG, Field KJ, Read DJ, Bidartondo MI (2018) Ancient plants with ancient fungi: liverworts associate with early-diverging arbuscular mycorrhizal fungi. Proc R Soc Lond B Biol Sci 285:1888

Schüßler A (2012) The *Geosiphon*–*Nostoc* endosymbiosis and its role as a model for arbuscular mycorrhiza research. In: Hock B (ed) The mycota IX. Fungal associations, 2nd edn Springer, Berlin, 77-91

Tedersoo L, Jairus T, Horton BM, Abarenkov K, Suvi T, Saar I, Koljalg U (2008) Strong host preference of ectomycorrhizal fungi in a Tasmanian wet sclerophyll forest as revealed by DNA barcoding and taxon-specific primers. New Phytol 180:479-490

Tedersoo L, Smith ME (2013) Lineages of ectomycorrhizal fungi revisited: Foraging strategies and novel lineages revealed by sequences from belowground. Fungal Biol Rev 27:83-99

Yamamoto K, Endo N, Degawa Y, Fukuda M, Yamada A (2017) First detection of *Endogone* ectomycorrhizas in natural oak forests. Mycorrhiza 27:295-301
